# Effects of Nonpharmacological Interventions on Disruptive Vocalisation in Nursing Home Patients With Dementia—A Systematic Review

**DOI:** 10.3389/fresc.2021.718302

**Published:** 2022-02-03

**Authors:** Saad Bilal Ahmed, Alfredo Obieta, Tamsin Santos, Saara Ahmad, Joseph Elliot Ibrahim

**Affiliations:** ^1^Department of Geriatrics, Ballarat Health Services Ballarat, Queen Elizabeth Centre, Ballarat Central, VIC, Australia; ^2^Department of Biological and Biomedical Sciences, The Aga Khan University, Karachi, Pakistan; ^3^Health Law and Ageing Research Unit, Department of Forensic Medicine, Monash University, Melbourne, VIC, Australia

**Keywords:** dementia, disruptive vocalisation, interventions, nursing homes, randomised controlled trial

## Abstract

**Background:**

Vocally disruptive behaviour is a common and difficult to treat condition in older residents with dementia. The aim of this systematic review is to evaluate the efficacy of nonpharmacological interventions in its management in persons with dementia residing in a nursing home.

**Methodology:**

A systematic search was conducted using Ovid MEDLINE, CINAHL, and Cochrane databases and reference lists from relevant publications on various nonpharmacological approaches to manage vocally disruptive behaviour in nursing home residents. The method of appraisal was through the National Institutes of Health scoring for the Quality Assessment of controlled intervention studies. Inclusion criteria included residents of nursing homes over the age of 65 with dementia and disruptive vocalisation. Only randomised controlled trials published in English were included.

**Results:**

A total of 5,606 articles were identified, which cover 501 trials, of which 23 were selected. There were fourteen studies observed to have an impact of clinical and statistical significance with interventions including (i) a multidimensional approach with different nonpharmacological interventions, (ii) multisensory stimulation, (iii) staff education and training, (iv) personalised bathing, and (v) pain recognition and appropriate management. Seven studies demonstrated no observable effect whereas two showed worsening in vocally disruptive behaviour.

**Conclusions:**

Many aspects of vocally disruptive behaviour management are poorly understood. Limited empirical evidence supports the use of several nonpharmacological interventions to reduce it. There is more robust evidence to support the use of a tailored approach to management over the universal approach.

## Introduction

Vocally disruptive behaviour is a group of behaviours that may or may not be aggressive, which include incoherent talking, repeated speeches, and creation of incomprehensible, incoherent noise as screams, shouts, moans, and chants (1, 2). It may be goal-orientated or aimless (3) or may be a response to unmet needs or demands and an attempt at communication by people who are unable to verbally express their needs or demands (4).

Although these figures are from 20 years ago and there has been a lot of improvements in dementia care since the 1990s through the person-centred care movement, it is anticipated that the prevalence of vocally disruptive behaviour will increase in the future as the number of people with dementia grows. Over half of the people living in nursing homes have dementia (5) and studies report the occurrence of vocally disruptive behaviour in nursing homes between 10 and 40% (6–10). Factors influencing the character of vocally disruptive behaviour include dementia, insomnia, confusion, loneliness, pain, anxiety, depression, psychosis, and narcissistic behaviour (3, 4, 11). Vocally disruptive behaviour typically worsens with the person's physical and mental deterioration (12) and is often a cause of concern for families and caregivers (13, 14).

Vocally disruptive behaviour management can comprise both pharmacological and nonpharmacological approaches (15). The rationale for nonpharmacological interventions being preferred as first-line management is because there is less risk associated than pharmacological treatment (16). Conversely, pharmacological treatments especially polypharmacy may have significant risk of harm from drug side effects and drug interactions and have limited efficacy (16, 17). To minimise the detrimental effects of polypharmacy, it is imperative to follow nonpharmacological treatment options for the management geriatric health issues (18, 19). Nonpharmacological approaches recognised for disruptive vocalisation management which include music, massage and touch interventions, and environmental manipulations that should be tailored according to the individual person's needs and circumstances (14, 20).

The objective of this review is to determine the effectiveness of nonpharmacological interventions in the management of disruptive vocalisation in persons with dementia residing in a care home with nursing.

## Methods

### Search Strategy and Eligibility Criteria

This systematic review was conducted in accordance with the Preferred Reporting Items for Systematic Reviews and Meta-Analyses (PRISMA-P) statement (21).

The review consisted of peer-reviewed literature published in English on or after the year 2005. The review was conducted in three phases.

The initial phase consisted of a limited search of the Ovid MEDLINE, CINAHL, PUBMED, and Cochrane databases to identify keywords contained in the title or abstract, and relevant MeSH headings and descriptor terms (Table 1). The second phase of the search was more extensive, using the appropriate keywords for each of these databases (Table 1). The final phase consisted of a bibliographic review of the selected articles.

**Table 1:** Search strategy concepts.

**Concept 1:** **dementia**	**Concept 2:** **disruptive vocalisation**	**Concept 3: nursing home institutionalisation**
1. Alzheimer's disease 2. Aphasia primary progressive 3. Binswanger encephalopathy 4. cadasil 5. corticobasal degeneration 6. delirium dementia amnestic cognitive disorders 7. dementia multiinfarct 8. dementia presenile 9. dementia senile 10. diffuse Lewy body disease 11. frontotemporal dementia 12. Kluverbucy syndrome 13. ‘mixed depression and dementia' 14. Multiinfarct dementia 15. pick disease of the brain 16. senile dementia 17. vascular dementia 18. alzheim* 19. Binswanger* 20. dement* 21. fronto?temporal or cortico?basal or fronto temporal or cortico basal or frontal lobe 22. posterior cortic* atroph* 23. dement*or alzheim* 24. cerad 25. subcortic* or subcortic* 26. encephalopathy* or leukoencephalopath* 27. mesulam 28. demen* or alzheim*	Behaviour management disrupt* behaviour disrupt* verbal* disrupt* vocal* echolalia inappropriate verbal* inappropriate vocal* repetiti* talking repetiti* vocal* verbal aggression verbal* agitat* verbal* behaviour* verbal* disrupt* vocal* agitat* vocal aggression vocal* behaviour vocal* repetiti* calling out call*	Nursing homes Homes for the aged assisted living facilities Long-term care Health services for the aged Skilled nursing facilities Housing for the elderly Residential facilities aged care Assisted living Convalescent home* Elder home Old people* home* Retirement facilit* Retirement home* Residential aged care RACS Residential elderly care facilit* Long-term care. Homes for the aged. homes for the elderly People* hom* old older or elderly or ageing Nursing hom* or facilit* or hostel or servic* or settin* or institution* Residential aged care servic* or settin* or facilit* or care facilit* or hous* or hom* or institution* Retirement facilit* or service* or settin* or institution* High care facilit* or servic* or settin* or institution* Intermediate care facilit* or servic* or settin* or institution* Low care facilit* or servic* or settin* or institution*

Results from the search strategy were exported into EndNote X8 software (Thomson Reuters). Duplicates were removed and the final set of articles was exported into the Covidence for screening. Covidence is the primary tool for screening and data extraction facilitating authors conducting standard intervention reviews. It is devised to operate functions such as upload search results, filter abstracts, complete manuscripts, perform comprehensive data assortment, calculate bias, resolve incongruities, and export data to Excel sheet for effective and well-organised review production. The extracted data items were then recorded in a Microsoft Excel 2013 spreadsheet.

Titles, abstracts, and reviewed full texts were screened by researchers SBA and SA and checked by JEI, and conflicts over inclusions were mutually discussed and solved by all the authors.

### Eligibility Criteria

#### Types of Studies

This review considered randomised control trials, as they provide experimental approach to reduce the bias.

#### Types of Participants and Setting

Participants were adults over 65 years, with dementia and a permanent resident in a nursing home. Permanent resident is a person who is living in a nursing home for 90 days (3 months) or longer.

Setting: Nursing home: “a nursing home is a facility with a domestic-styled environment that provides 24-h functional support and care for persons who require assistance with activities of daily living and who often have complex health needs and increased vulnerability. Residency within a nursing home may be relatively brief for respite purposes, short term (rehabilitative) or long term, and may also provide palliative or hospice and end-of-life care. In general, most nursing homes also provide some degree of support from health professionals” (22). Terms used to describe these settings include nursing home, residential aged care facility, retirement home, care home, care home with nursing, domiciliary facility, long-term care, and assisted-living facility (22).

Dementia: Cognitive impairment occurs with the problems in thought process affecting learning disabilities, concentration difficulties, reductions in mental functions and mild cognitive impairment which is usually a stage between cognitive decline and dementia. Dementia is broadly defined as a wide range of gradual but progressive mental disabilities that impair memory, effect personality changes, and reduce reasoning (23). It is often complicated by advanced ageing with high degree of comorbidity.

Exclusion: Where the study focus is entirely on community-style accommodation such as family home, private home, private-shared living, and community-based living facility. Studies published in a language other than English and nonpharmacological management of vocally disruptive behaviour were excluded from the review.

#### Outcomes

The outcome measure was the level and nature that is the duration, intensity, and disruptiveness of vocally disruptive behaviour and entails both vocal aggression and vocal nonaggression. In vocal aggression, there is use of language, which is harsh, abusive, intimidating, indecent, or disrespectful; this may include cursing of others (6, 24–26).

In vocal nonaggression, older people make sounds that include crying, wailing, perpetual demands for care and assistance, retelling expressions, and voicing and gibberish speaking, chattering, singing, and humming.

#### Data Extraction

Sixty-three full text articles exported to Covidence (a software management programme for systematic reviews) were assessed for eligibility. Of the 23 eligible articles, the following information was extracted by SBA and SA: study, author, year and country, type of home care, ethnicity, study characteristics, intervention, study period, number of participants, sex, mean age, assessment tools, and outcome on vocalisation. The results were checked by JEI.

#### Quality Assessment

Risk of bias was assessed using the National Institutes of Health reference tool for quality assessment of controlled intervention studies [(27), https://www.nhlbi.nih.gov/health-topics/study-quality-assessment-tools] because it is a complete, practical, convenient, and validated state-of-the-art methodology (Table 2).

**Table 2:**
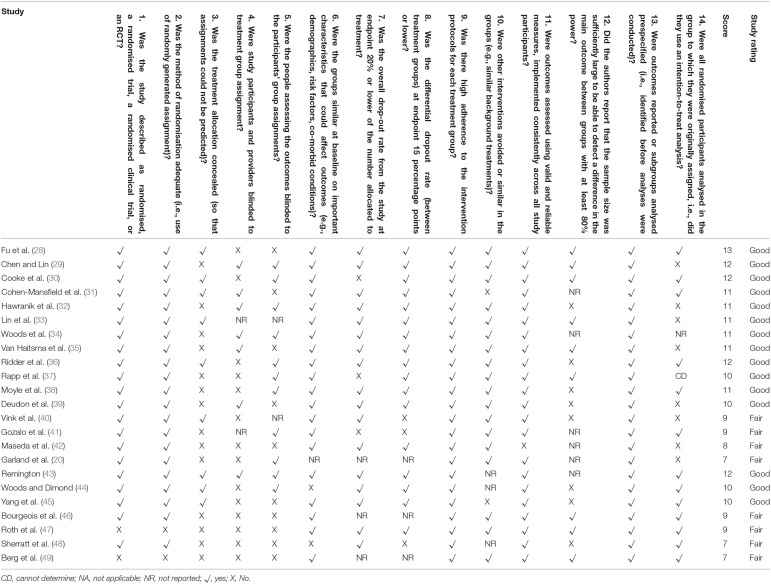
Quality appraisal of included studies through National Institutes of Health scoring for controlled intervention studies.

#### Data Analysis

Due to the diversity of the studies, the meta-analysis was not conducted, and a systematic review approach of a narrative review was adopted.

## Results

### Study Selection

**Figure 1:**
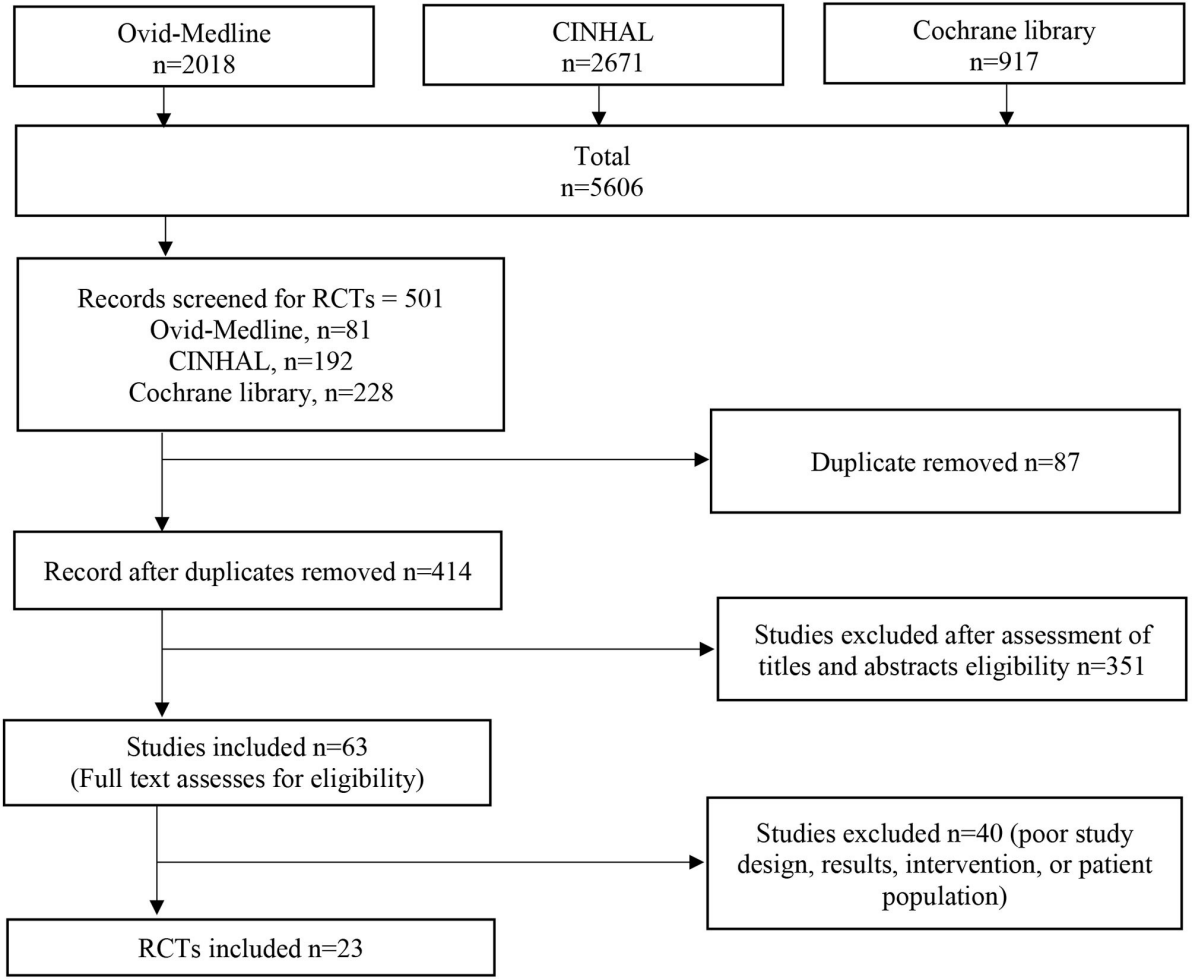
Flow chart of literature search.

Of the 5,606 studies initially found, there were 501 randomised controlled trials, of which 23 underwent review. Figure 1 outlines the process used for finding relevant trials.

#### Quality of Included Studies

Table 2 shows the randomised control trials included in this review with their appraisal score.

Overall, 15 studies were rated as “good” (quality rating appraisal range 10–14) and eight were rated as “fair” (quality rating appraisal range 5–9).

#### Study Characteristics

The studies were carried out in Australia (*n* = 4) and the United States (*n* = 7), Taiwan (*n* = 3), England (*n* = 2), Canada (*n* = 1), the Netherlands (*n* = 1), Spain (*n* = 1), Sweden (*n* = 1), Denmark and Norway (*n* = 1), France (*n* = 1), and Germany (*n* = 1). All studies were published between 2005 and 2015.

A combined total of 2,275 residents were included across 23 studies, with 1,325 nursing home residents in intervention groups and 950 nursing home residents in control groups. The overall mean age was 83.9 years, with an intervention group mean age of 84.0 years and a control group mean age of 83.7 years. Overall, 74.3% of participants were women; women made up 70.1% in intervention groups and 72.4% in control groups. Participants were of mixed ethnicity. Supplementary Table describes the randomised controlled trials included in the review with year of publication, sample size, country, and length of observation for each study.

### Study Findings

Vocal aggression and nonaggression may be either coherent or incomprehensible. In most of the studies, the terms used were as follows:

1. verbal agitation, verbal aggression, and verbalisation (28–30, 35, 39, 43–49).
2. Verbal aggressive and nonaggressive (33, 37, 44–49).
3. Disruptive vocalisation and screaming (34, 36, 47).
4. Verbal aggression or agitation consisting of swearing, cursing (20), constant requests for attention, shouting, negativism, complaining, repetitious sentences or questions, calling for help, protesting, yelling, and screaming (32, 41, 42, 46, 47, 49, 50).
5. Verbally nonaggressive agitation comprises of repetitive questions, constant requests, and coherent verbalisations (20, 35, 46, 49, 50).

Vocally disruptive behaviour was determined in the residents with dementia. Dementia was defined in a variable manner, and different scales were used in the selected randomised controlled trials:

1. Dementia defined as mini-mental state examination score of 24/30 or less, and features of Alzheimer's disease according to the American Psychiatric Association Diagnostic and Statistical Manual of Mental Disorders, Fourth Edition or International Classification of Disease, Tenth Edition was used in seven studies (28, 30, 33–35, 39, 40).
2. Mini-Mental state examination was used as the tool for diagnosis of dementia in only nine studies (20, 29, 31, 32, 37, 38, 46, 47). However, there was a variation in mini-mental state examination scores from 18 to 25 as a cut-off point.
3. Cognitive Performance Scale was used in another study (41).
4. Two studies used nursing homes' records (36, 42).

### Types of Interventions

Of the 23 randomised controlled trials, five were single-blind, three were double-blind, five were crossover, and there were single trials of attention control, block randomisation, and cluster randomised controlled longitudinal trials.

#### Types of Outcome Measures

Functional status and outcome measures of most of the studies were analysed through objective-based inventories that involved the Cohen-Mansfield Agitation Inventory Scale or its modified versions (7, 20, 28–30, 32, 33, 36, 37, 39, 40, 42–45, 51). The other less-frequently used inventories included Mini-Mental State Examination (28, 32, 34, 46–48), Verbal Descriptor Scale (29), Pain Assessment in Advanced Dementia Scale (29), Rating Anxiety in Dementia Scale (30), Agitation Behaviour Mapping Instrument (31), Lawton's Modified Behaviour Stream, Intervention logs (31), Quality of life (Alzheimer's disease-related quality of life) (36), Observed Emotion Rating Scale (38), Observation Scale score (39), the Neuropsychiatric Inventory–Nursing Home (42) and Brief Agitation Rating Scale (44), and the phenomenological-hermeneutic analysis method (49).

Subjective assessments included use of videotaped observation of participants' activities, resident medical records, memory and behaviour checklist (34), direct observations in the form of 10-min ‘behaviour streams' (35), verbal agitation behaviour factor (42), and secondary measures such as bath duration, bath modality, and use of antipsychotic medication (41).

### Adherence to Interventions

Eleven studies (20, 28–34, 36–39, 41–49) showed high level of adherence to intervention which refers to the degree to which participants correspond to the intervention assigned to them including trial retention and adherence to the follow-up protocol of procedures and assessments.

### Outcomes of the Various Nonpharmacological Interventions

#### Music

Six randomised controlled trials assessed the potential benefits of music therapy. Three studies showed reduction in vocally disruptive behaviour with programmes that included individualised music therapy by professionally trained music clinicians (36, 43) and group music therapy with slow tempo instruments, glockenspiel, and specially selected music interventions (33). Another study contradicted these results, as vocally disruptive behaviour increased with a live music programme, song singing, and listening (30), whereas no effects of live music were seen in vocally disruptive behaviour (48). Another study with group music therapy and general recreational activities showed a short-term decrease in the behaviour but no additional benefit over standard recreational activities (40).

#### Massage

**Figure 2:**
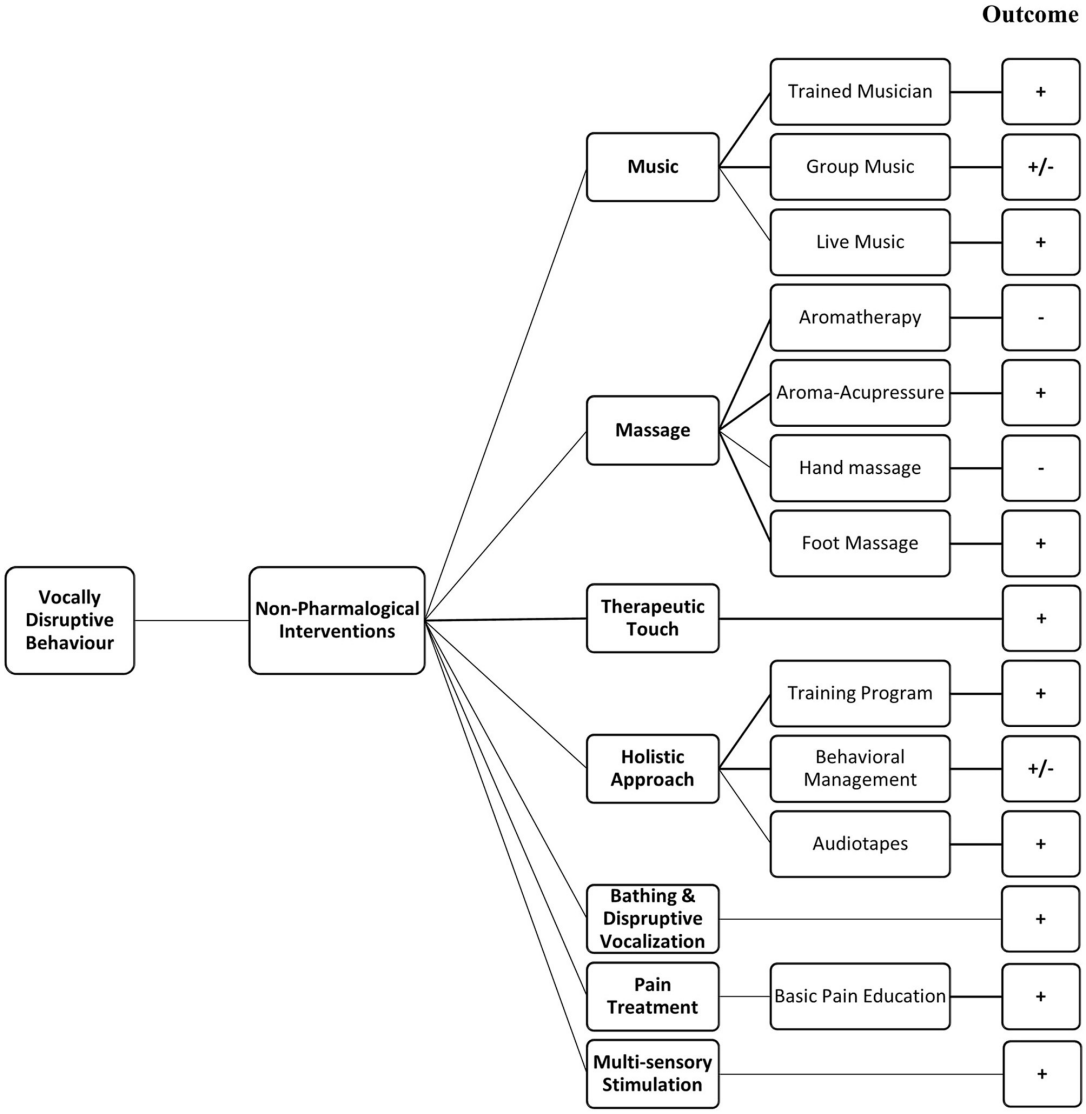
Summarised effects of non-pharmacological interventions. +, Positive response; –, negative response; +/–, variable response.

Aromatherapy using lavender oil alone or combined with hand massage had no significant benefit on vocally disruptive behaviour upon Cohen-Mansfield Agitation Inventory evaluation (28). Aroma-acupressure found to exert greater effects than aromatherapy (45). No effects of hand massage were seen on aggressive behaviour in another study (43). A foot massage vs. quiet presence of study (38) suggested that foot massage increased vocally disruptive behaviour, whereas the observed emotional rating scale showed increased alertness in the foot massage group (Figure 2).

#### Therapeutic Touch Therapies

In therapeutic therapy practice, the therapist places their hands on or near their patient's body with the intention of helping or healing by focusing on balancing the total energies of the person and boosting the body's natural healing ability rather than on the treatment of specific physical diseases. This is based on “the conscious use of the hands to direct or modulate, for therapeutic purposes, selected nonphysical human energies that activate the physical body” (52).

In a Canadian study (34), the intervention was given two times daily for 5-7 min for 3 days; this significantly reduced vocally disruptive behaviour in an experimental group compared with placebo therapeutic touch and usual care groups. Another study supported the results that significant reduced agitated behaviour was seen by 3 days intervention for 5–7 min (44). In another study in North America (32), therapeutic touch was given once daily for 5 days; however, there was no observed effect on the vocally disruptive behaviour.

#### Multicomponent (Holistic) Approach to Management of Agitation

Seven randomised controlled trials had a multicomponent approach for vocally disruptive behaviour management in nursing home residents. Overall, the nonpharmacological interventions were focused on fulfilling unmet needs in the older residents, such as boredom or pain, to significantly reduce the behaviour (31). Two studies that analyse a staff education and training programme indicated a significant decrease in the vocally disruptive behaviour (39, 47). The behaviour management programme for caregivers was also successful as it improved the perception to manage the patient's behaviour (46). However, no significant effect was found on the vocally disruptive behaviour in nursing home residents with dementia in a study with a complex guideline-based intervention (37).

Researchers in one Australian study (20) observed nursing home residents after multiple exposures to 15-min audiotapes of simulated family presence, preferred music, or a placebo (reading from a horticultural text) and noted significantly reduced the vocally disruptive behaviour with simulated presence, but not music. Berg et al. (49) disclosed the strategy to manage the patient's behaviour by conducting the interviews of 13 nurses in dementia ward. Likewise, Van Haitsma et al. (35) observed the effectiveness of an individualised positive psychosocial intervention of preferred one-to-one based activities and standardised one-to-one activities. It was observed that the intervention group experienced reduced the vocally disruptive behaviour, greater alertness, and pleasure when compared to the standardised activity group.

#### Bathing and Disruptive Vocalisation

In a North American study, the goal of bathing without conflict was assessed by provision of in-bed baths and reducing average bath duration; the vocally disruptive behaviour was significantly reduced (41).

#### Pain Treatment

In a study from Taiwan, it was observed that providing basic pain management education and implementing a pain recognition and treatment protocol in comparison with only basic pain education resulted in a significant decline in the weekly average scores with the verbal descriptor scale. The pain protocol was followed by nonpharmacological and pharmacological management, monitoring effectiveness of treatment, and found to be effective for nurse-led pain management of nursing home residents (29).

#### Multisensory Stimulation

In a study from Spain (42), multisensory stimulation through exposure to Snoezelen room with various elements to stimulate the visual, olfactory, tactile, and auditory senses showed a decrease in the vocally disruptive behaviour.

## Discussion

To our knowledge, this is the first systematic review to examine the nonpharmacological management of the vocally disruptive behaviour exclusively in older care home residents with dementia and cognitive impairment. Twenty-three randomised controlled trials are included in this review. Fourteen studies reported a statistically significant decrease in the vocally disruptive behaviour with a nonpharmacological intervention (20, 29, 31, 33, 34, 36, 39–41, 44–47), two studies noted worsening of vocalisation (30, 35), and seven studies did not observe any change (28, 32, 37, 38, 43, 48, 49), whereas other six studies showed significant reduction in the vocally disruptive behaviour during intervention and also after follow-up (29, 33, 39, 42, 45, 46). These studies used a relatively larger number of participants, rigorous delivery of nonpharmacological interventions, a medium- to long-term follow-up period, and interventions useful across cultural settings (Supplementary Table).

Supported by the previous evidence and through this review, it is inferred that the best evidence emerges for interventions that are individualised diagnose and manage the vocally disruptive behaviour, but the ideal approach may vary depending on the age, level of dementia severity, and cultural background of the nursing home residents, and also availability of skilled staff (41, 53–55).

In this review, we endeavoured to study a homogenous population by including studies involving only older nursing home residents with dementia who have used nonpharmacological interventions. Although it is difficult to observe the effectiveness of nonpharmacological interventions in persons with dementia (53, 56), the different nonpharmacological approaches commonly used for the purpose are as below.

For music therapy, variable results were found. In a previous review, a nonsignificant effect on disruptive vocalisation was noted with soothing music, which was possibly due to perception of music as a noxious stimulus (43). Likewise, the effects of live music and group music interventions did not demonstrate significant therapeutic value as music predominates individual preferences, traditional behaviour, and also duration of sessions (30, 48). However, two separate examinations also revealed a reduction in disruptive vocalisation with music therapy during intervention (55) and even after cessation of intervention (33). Hence, music therapy can be safe, inexpensive, and acceptable way to treat disruptive vocalisation in clinical settings.

The outcomes of the intervention of hand and foot massage with aromatherapy have been diverse with contrary effects on disruptive vocalisation. Moyle et al. (38) found nonsignificant effects of foot massage on agitation in persons with dementia. However, these results were inconsistent with the pilot study in which the therapy was given by well-trained staff (38). Therefore, the nonsignificant results might be the reason of unfamiliar participants (38). Similarly, another study was performed by Fu et al. (28), based on aromatherapy, and hand massage showed nonsignificant effects on the behaviour. This may be due to the olfactory dysfunction often seen in people with dementia causing contrary effects (28). In contrast, some reports suggested a decline in the vocally disruptive behaviour with massage therapies (44, 45). Aroma-acupressure has also presented the significant decline in vocally disruptive behaviour in patients with dementia and the effects were greater than aromatherapy (45).

One systematic review showed a significant reduction in the vocally disruptive behaviour with professional hand massage (32), whereas other study that performed by Remington suggested no effect on the behaviour with light-pressure hand massage. It was also proposed that proper training was required for the better outcomes of the intervention (43). These mixed results are therefore insufficient to support massage therapy at this point.

A multicomponent approach appears to be the most effective way of managing the vocally disruptive behaviour. This includes multiple nonpharmacological interventions, staff training, and simulated family presence through audiotape. Individualised interventions and also tailored approaches showed a significant decrease in the vocally disruptive behaviour (31, 35, 39), and simulated family presence improved the vocally disruptive behaviour (20). Similar findings were reported in previous reviews (46, 55, 57) and work of other authors (46–48, 53, 54).

Overall, it is seen that an individualised and targeted approach is most likely to be successful in reduction of the vocally disruptive behaviour. Comprehensive assessment with individualised and tailored multicomponent interventions is likely to have the most significant effect. This may be resource intensive, but there is a greater chance of significant and wide-ranging impact compared with single interventions.

Pain recognition and treatment includes primary and secondary pain assessment, management, and regular reassessment of nursing home residents with dementia. These approaches showed results similar to other studies (49, 58), as summarised in a previous review (55). However, very limited research has explored the role of pain management in behaviour changes in residents with dementia (59). However, it is not always possible to measure the impact of pain management in residents with dementia due to limitations in accurately assessing pain. Other potential barriers to wider use of pain management strategies are adverse effects of analgesics, duration of treatment, and difficulty in monitoring the improvement in pain levels.

Changes in the vocally disruptive behaviour outcomes may vary for community-dwelling individuals compared with nursing home residents due to variables such as living close to loved ones and being at home. Hence, music, massage, and pain relief interventions may be effective depending on the residential circumstances of study participants. A multisensory stimulation study with a small number of nursing home participants reported a significant reduction in the vocally disruptive behaviour (41) and was consistent with recent reviews (53–55).

The use of psychotropic medication to manage the vocally disruptive behaviour in older adults with dementia is limited by idiosyncratic response to medication and the potential for medication to harm; therefore, research into nonpharmacological interventions should be a priority (60, 61).

The present review summarises the importance of nonpharmacological interventions given for management of vocally disruptive behaviour in older adults. The therapies identified to have considerable effects on disruptive behaviour included music, therapeutic touch, acupressure, and aromatherapy. However, heterogeneity of the geriatric population distribution indicates a complex link between disease severity and management which requires further research on the nonpharmacological interventions for better understanding of the outcomes.

## Strength and Limitations

This review includes important randomised controlled trials with the use of comprehensive search terms for vocalisation. Major limitations were that the selected articles were chosen from the time period from January 2005 to December 2019, published only in English, and indexed in one of the three common search engines (Ovid, CINHAL, Cochrane). It is possible that the studies in other languages and those published before 2005 may have relevant information.

The lack of consistently used definitions of both vocally disruptive behaviour and dementia in the studies may have led to inconsistencies in reported results.

### Generalisability

Of the 16 randomised controlled trials in this review, four were conducted in the United States and Australia. Since different countries vary in socioeconomic factors, demographics, and health care systems, caution is required when generalising the results to other countries (62).

## Future Research

Conducting clinical research in residents with dementia is complex, predominantly due to ethical issues, difficulty in obtaining informed consent, recruiting residents, and capturing data. Comprehensive strategies are required to overcome these limitations (63) with uniform clinical tools to define and categorise vocally disruptive behaviour for standardisation of outcome measures and better comparisons between nonpharmacological management studies. Collaborative research with large scale, multicentre trials may be the best approach to gather empirical evidence for vocally disruptive behaviour management. Hence, further research into nonpharmacological interventions for vocally disruptive behaviour should be a priority.

## Conclusions and Implications

Nonpharmacological interventions in the vocally disruptive behaviour are heterogenous and generally safe approach to management, although the guidelines and recommended approach begins with thorough bio-psycho-social assessment; however, variability of the results may occur because of the number, length, content and intensity of sessions, and recommendation of approaches and techniques. Despite the limited evidence, these strategies are potentially useful adjuncts in vocally disruptive behaviour management, and individually tailored interventions may alleviate vocally disruptive behaviour in older nursing home residents with dementia.

Despite the range of research studies, no clear guidelines emerge for disruptive vocalisation management for clinicians, nurses, and carers in nursing homes. Based on the available evidence, a multisensory approach with a combination of different nonpharmacological interventions, staff training, pain recognition and treatment, and person-centred care plans with personalised interventions is supported by reasonably strong evidence. Individualised or group music therapy has variable results and is worth trialling in individual circumstances. The widespread application of therapeutic touch, massage, and aromatherapy is currently unsupported.

## Data Availability Statement

The raw data supporting the conclusions of this article will be made available by the authors, without undue reservation.

## Author Contributions

SB: conceptualisation, methodology, and original draft preparation. AO: data curation, writing, and critical reading. TS: investigation, study design, and writing and critical reading. SA: visualisation, validation, writing—reviewing, and editing. JE: supervision, study design, result interpretation, formal analysis, and critical reading. All authors contributed to the study and have approved the final draft of the manuscript.

## Conflict of Interest

The authors declare that the research was conducted in the absence of any commercial or financial relationships that could be construed as a potential conflict of interest.

## Publisher's Note

All claims expressed in this article are solely those of the authors and do not necessarily represent those of their affiliated organizations, or those of the publisher, the editors and the reviewers. Any product that may be evaluated in this article, or claim that may be made by its manufacturer, is not guaranteed or endorsed by the publisher.
