# Supplementary material for: Effects of Nonpharmacological Interventions on Disruptive Vocalisation in Nursing Home Patients With Dementia—A Systematic Review
Source: Front Rehabil Sci. 2022 Feb 3;2:718302. doi: 10.3389/fresc.2021.718302 (PMC9397760; doi:10.3389/fresc.2021.718302)
Supplement: Supplementary file 1 [file Table_1.pdf]

Supplementary table: Randomised controlled trials included in the review with intervention and appraisal score.

Study author and study type/design	Settings	Aim	Country	Intervention	Intervention period/study period and follow up (where provided)	Population and sample size	Assessment	Outcome/Effect on disruptive vocalisation	Appraisal score
Fu et al, (2013)<br><br>Single blinded RCT	Three Long term care facilities	To investigate the effect of aromatherapy (3% lavender oil spray) with and without hand massage on disruptive behaviour	Australia	Aroma therapy and hand massage, delivered by the facility staff.	Intervention period was for 6 weeks. Post-tests follow up assessment of aggression with Cohen-Mansfield Agitation Inventory was done after 6 weeks of completion of intervention was done.	67 residents, male=41%, female=59%, with a mean age of 84 years	1) Cohen-Mansfield Agitation Inventory 2) Mini-Mental State Examination	Kruskall-Wallis H tests showed that none of the interventions significantly reduced disruptive behaviour at the end of the intervention nor during the post-test follow up (p < .05).	13
Chen and Lin (2015)<br><br>A double-blind cluster randomised controlled trial	Six dementia special care units	To compare the effect of a Pain Recognition and Treatment protocol coupled with basic pain education (experimental group) versus basic pain education alone (control group)	Taiwan	Basic Pain education: Pain Recognition and Treatment, delivered by health care providers	6 hours of basic pain education and 4 hours of instruction in the use of the protocol.<br>Study period: 3 months (Sep 2012-Nov 2013) with a 3month follow-up	195 residents, male=50%, female=50%, with a mean age of 82.7 years	1)Verbal Descriptor Scale, 2) Cohen-Mansfield Agitation Inventory	Significant reduction in weekly average scores on the verbal disruption at both postintervention and the 3-month follow up. The scores for the verbal disruption are not provided in the paper.	12

Cooke et al, (2010)<br><br>A randomised cross-over design	Two aged care facilities	To investigate the effects of participation in group music programme, involving facilitated engagement with song-singing and listening, on agitation and anxiety	Australia	1) music 2) reading, delivered by aged care staff	40-min live group music programme, three times a week for eight weeks Study period: October 2008 to March 2009 (6 months)	47 residents, male=29.8% female=70.2%, with a mean age of 87.2 years	1) Cohen-Mansfield Agitation Inventory – Short Form, 2) Rating Anxiety in Dementia Scale	A significant increase in the frequency of verbal aggression over time, regardless of group ($F(2,46)= 3.534$, $p<0.05$).	12
Cohen-Mansfield et al, (2012)<br><br>A randomised, placebo-controlled clinical trial	9 nursing homes	to determine the efficacy of nonpharmacologic individualized interventions (individualized to address unmet needs such as boredom or pain) in decreasing agitation	USA	Non pharmacologic interventions, Treatment Routes for Exploring Agitation, delivered by research assistant	Treatment routes for exploring agitation for 2 weeks<br>Study period:5.5 years (June 2006 until December 2011)	125 residents, (n = 89) and a placebo control group (n = 36), with a mean age of 85.7 years	1)Agitation Behaviour Mapping Instrument 2)Videotaped 3)Lawton's Modified Behaviour Stream.	Significant decline ($p<.01$) in verbal agitation, physical nonaggressive and significant increase in pleasure and interest.	11
Hawranik et al, (2008)<br><br>A randomised multiple time series, blinded, experimental design	Special needs units of one long-term care facility	To determine the preliminary evidence for the potential for therapeutic touch in dealing with agitated behaviours	USA	Intervention was given by the resident's nurse, volunteer or practitioner	4 weeks.<br><br>Follow up after 24 hours postintervention and 2 weeks after final intervention was done.	51 residents, female=36, male=15, with a mean age of 82.8 years	1) Cohen-Mansfield Agitation Inventory 2) Mini-Mental State Examination 3) Resident medical records 4) Intervention logs	No significant differences in physically aggressive ($p = .32$) and verbally agitated behaviours ($p = .37$) during intervention observed across the three study groups. Also, no significant difference observed during postintervention	11

								follow-up in physically aggressive behaviours (p = .51) and verbally agitated behaviours (p=.21) across the three study groups.	
Lin et al, (2011)<br><br>Permuted blocked randomisation experimental study	3 nursing home facilities	To explore the effectiveness of group music intervention against agitated behaviour	Taiwan	Music vs normal daily activities, delivered by the trained psychologist, researcher (therapist)	Twelve 30-min group music sessions, twice weekly for six weeks. Three follow assessments were performed at the 6th and 12th group music sessions and at 1 month after cessation of the intervention.	104 residents, Experimental group n=49 male=46.94%, female=53.06% with a mean age of 81.46 years; and Control n=51 male=47.06%, female=52.94% with a mean age of 82.15 years	Chinese Version of the Cohen-Mansfield Agitation Inventory	Statistically significant decrease in verbally non-aggressive behaviour in the experimental group at three time-point comparisons versus pretest: at 6th session (p=0.042), at 12th session (p= 0.010) and at 1 month after cessation of the intervention (p= 0.037). A significant decrease in verbally aggressive behaviours in the experimental group at 6th session versus pretest (p=0.021),	11

								and nonsignificant reduction at 12th session (p= 0.104) and 1-month postintervention (p= 0.764) was observed.	
Woods et al, (2005)<br><br>Randomised, double blind, three-group experimental study	Three special care units within three long term care facilities	To examine the effect of therapeutic touch on frequency and intensity of behaviour symptoms of dementia	Canada	Therapeutic touch 3 groups, 1) therapeutic touch (experimental) 2)Placebo therapeutic touch (placebo) 3) Usual care (control); delivered by trained research team.	Therapeutic touch given twice daily for 3 days. Preintervention for 3 days, intervention for 3 days and a post intervention follow up for 3 days immediately after intervention.	57 residents, male=19%, female=81%, with a mean age of 81.04 years	1) Memory & Behaviour check list<br>2) Mini-Mental State Examination	Intervention produced a significant decrease in vocalization (p=0.34). A significant decrease in overall behavioural symptoms of dementia and vocalization from preintervention to postintervention was also observed (p=.025).	11
Van Haitsma et al, (2013)<br><br>Randomised controlled study	A large non-profit nursing home divided into eight nursing home units	To study the effectiveness of individualized activities, led by certified nursing assistants, to increase positive and reduce	USA	Individualized activities led by certified nursing assistants. Group assigned to 1) Usual care 2) a) Attention control b) Individualized Positive Psychosocial	3 weeks treatment period with 2 months of training of research assistant prior to intervention	180 residents, female=82.2%, male=17.8%; with a mean age of 88.7 years	Direct observations in the form of 10-min "behaviour streams."	Attention control group displayed more anger and very negative verbal behaviour (p= .2855) as well as uncooperativeness than usual care or Intervention;	11

		negative affects and behaviour		Intervention; delivered by staff members of the nursing homes.				delivered by staff members (p= .0167).	
Ridder et al, (2013)<br><br>Pragmatic, two-armed, cross- over, exploratory, randomised controlled study	14 nursing homes, 4 in Norway and 10 in Denmark	To examine the effects of individual music therapy on agitation, and to explore its effect on psychotropic medication and quality of life.	Denmark & Norway	Individual music therapy or standard care, delivered by clinicians with approved university training in music therapy	Biweekly over a period of six weeks, altogether 12 sessions; total 14 weeks	42 residents, female=69%, male=31%, with a mean age of 81 years	1) Cohen-Mansfield Agitation Inventory 2) Alzheimer's Disease-Related Quality of Life	Six weeks of music therapy significantly reduced agitation disruptiveness (p= 0.027)	12
Rapp et al, (2013)<br><br>Cluster randomised controlled trial with blinded assessment of outcome in 18 nursing homes in Berlin and Germany,	18 nursing homes	To test the effect of a complex guideline-based intervention on agitation and psychotropic prescriptions	Germany	The guidelines of the American Geriatrics Society and the American Association of Geriatric Psychiatry were followed 1) Training of Nursing Home Staff 2) Nonpharmacological Interventions<br>3) Optimization of Pharmacological Interventions; delivered by the nursing home staff	10 months	304 residents, female=72.7%, male=27.3%; with a mean age of 81.56 years	Cohen-Mansfield agitation inventory	Aggressive behaviour decreased in invention group but no significant effect was observed on physical non-aggressive behaviour (p=.977) and verbally agitated behaviour (p= .357).	11

Moyle et al, (2014)<br><br>A randomised controlled trial using computer program (thus allocation concealment) within-subjects, crossover design with each subject serving as his/her own control	Five long-term care facilities	To compare the effect of foot massage (intervention) and quiet presence (control) on agitation and mood in people with dementia.	Australia	Foot massage vs quiet presence delivered by the research assistants.	10-min foot massage (intervention) or quiet presence (control), every weekday for 3 weeks	55 residents, female=66%, male=36%; with a mean age of 86.5 years	1)Cohen-Mansfield Agitation Inventory 2) Observed Emotion Rating Scale	No effect of the foot massage observed. Increase in verbal aggression subscale in both groups observed, but more so in the quiet presence than the foot massage group (p=0.03).	11
Deudon et al, (2009)<br><br>Randomised controlled trial	16 nursing homes	To evaluate the effectiveness of a staff education intervention to manage BPSD	France	8 weeks for staff education and training	The study conducted from 15 October to 15 December 2007, with the follow-up at 3 months being performed in March 2008	306 residents, male=68 female=238, with a mean age of intervention group 86.5 years and of controls 86 years.	1) Cohen-Mansfield Agitation Inventory and 2) Observation Scale score	Significant decrease in verbally non-aggressive behaviour ($p < 0.001$) in the intervention group.	10
Vink et al, (2012)<br><br>Randomised controlled design	Six Dutch nursing homes	To compare the effects of music therapy with general recreational day activities in reducing agitation	Netherlands	Music therapy with general recreational day activities; delivered by trained music therapist.	Music therapy and general activities were offered twice weekly for 4 months	77 residents, female=54, male=23, with a mean age of 82.16 years	Modified Cohen-Mansfield Agitation Inventory	Short term non-significant decrease in agitated behaviours from 1 h before to 4 h after each session (p= 0.090). This decrease was greater in the music therapy group than in the	9

								general activities group.	
Gozalo et al, (2014)<br><br>A randomised crossover diffusion study	6 nursing homes	To evaluate the effectiveness of the Bathing Without a Battle intervention in reducing physical and verbal aggressive behaviours for nursing home residents with dementia.	USA	Bathing Without a Battle intervention	Train the trainer model. Two stages stage 1: two-day joint training session Stage 2: Up to 2 months<br>Study period: 2 years 2009 to 2011.	240 residents, female=65.8%, male=34.2%, with a mean age of 84.7 years	1)Care Recipient Behaviour Assessment 2) secondary measures a) bath duration, b) bath modality c) antipsychotic medication use.	Significant reduction in verbal behaviour (p=.008) and significant decline in the combined verbal and physical behaviours<br><br>declined (p=.004)	9
Maseda et al, (2014)<br><br>Controlled longitudinal study with random allocation of the patients	A specialized elderly centre	To evaluate the positive effect of multisensory stimulation environment on neuropsychiatric symptoms in older people with dementia.	Spain	3 groups: 1) Multisensory stimulation environment 2) Individualized activities 3) Control. The intervention was delivered by trained occupational therapist.	Two 30-minute weekly individualized intervention sessions over 16 weeks with 8week postintervention follow up.	30 residents, female=27, male=3, with a mean age of 87.3 years	1) Cohen-Mansfield agitation inventory, 2) verbal agitated behaviour factor, and 3) Neuropsychiatric Inventory–Nursing Home	Significant time effects<br><br>in both groups in the Cohen-Mansfield agitation inventory total score (p<.049)<br><br>and verbally agitated behaviour (p<.001), with<br><br>an improvement in the level of agitation during the intervention.	8

									However, no significant differences were found between the groups. When comparing the MSSE group and the control group, a significant decrease in verbally agitated behaviour was observed from the pretrial to the posttrial in both groups (p=.012), with no significant intergroup differences	
Garland et al, (2013)<br><br>Randomized single blind crossover trial	Nine nursing homes	To compare the effectiveness of two individualized psychosocial treatments in reducing the frequency of physically and verbally agitated behaviors	Australia	Simulated family presence, music and placebo (reading from a horticultural text). Intervention was delivered by trained psychologists and nurses.	Fifteen-minute audiotapes of simulated family presence and preferred music were compared with a neutral audiotape (placebo) and usual care. Total observa-tion period lasted for 45 minutes each. 4 weeks	30 residents, F=63% M=37%, with a mean age of 79 years	Cohen-Mansfield agitation inventory	Simulated presence (p=0.037), placebo tape (p= 0.030) but not music (p= 0.101) resulted in significantly reduced counts of verbally agitated behaviors during treatment when compared with usual treatment. Likewise, simulated presence and preferred music both proved effective in reducing counts of physically agitated behaviors	7

Remington, (2002)<br><br>Single blinded RCT	four long-term care facilities	To observe the efficacy of calming music and hand massage on agitated behavior in persons with dementia.	USA	calming music and hand massage	Two weeks.<br><br>Participant received i) calming musing ii) hand massage iii) calming music and hand massage for 10 minutes and compare with iv) control with no intervention Cohen-Mansfield Agitation Inventory was used to record agitated behaviours.	The sample size was 68 with mean age of 60 years, comprising of 59 females, and nine males	Cohen-Mansfield Agitation Inventory	The effects were increased up to one hour following the intervention (F = 6.47, p<.01) which were similar for each group of intervention.<br>None of the interventions significantly affected to reduced physically aggressive behaviours (F = 1.93, p=.09), however, physically nonaggressive behaviours reduced in each group (F = 3.78, p< 01). No additive effects were found from simultaneous exposure to calming music and hand massage. At one hour following any intervention, reduced verbally agitated behaviour found. The scores for the verbal disruptive behaviour reduction were not	12

								provided in the paper.	
Woods, & Dimond, (2002)<br><br>Single blinded RCT	special care unit	The effects of therapeutic touch on agitated behavior and cortisol in persons with Alzheimer's disease.	USA	Therapeutic touch	The treatment period was three days. Therapeutic touch was given by PI. Post treatment assessment was done by Agitated Behavior Rating Scale.	Ten subjects who were 71 to 84 years were included comprising of 30% male and 70% females.	1. Brief Agitation Rating Scale.<br><br>2. Cohen-Mansfield Agitation Inventory	Reducing effects were seen in agitation and cortisol level in patients. The overall reducing effects in agitated behaviour was found $F(1,5)= 6.82$, $P = 0.00$.	10
Yang et al., 2015<br><br>Single blinded RCT	Three Long term care facilities	To compare aroma-acupressure and aromatherapy with respect to their effects on agitation in patients with dementia	Taiwan	Aroma-acupressure and aromatherapy	Intervention period was 4 weeks. Pre- test and post-test assessment of Cohen-Mansfield Agitation Inventory and heart rate variability.	186 subjects included comprising: Male= 73;<br><br>Female=26	The Cohen-Mansfield Agitation Inventory (CMAI) scale was used.	Aroma-acupressure had a greater effect than aromatherapy on agitation in patients with dementia. Significant differences were found in CMAI score in post-test and post three weeks ($p<0.01$).	10

Bourgoius. 1997	local support groups	To evaluate the efficacy of written cuing and behaviour management intervention given to care givers for reduction of repetitive verbalization	USA	12 weekly home visits to implement behavior management programs (written cuing) in response to repetitive verbalizations.	Caregivers were trained to respond systematically to patient by written cuing (index card, memory book page, or memo board).	7 caregivers were selected for trained about behaviour managing program.	Mini Mental State Exam	Improved perceptions of trained caregivers in managing patient behavior at the 3-month follow-up assessment. The significant effects were seen as treatment phase frequency was lower than baseline, $F(1,6) = 8.83$, $p < 0.05$. no significant effects were seen in follow-up and treatment	9
Roth et al., 2002	two nursing homes	To evaluate timed-event sequential analysis of agitation in nursing home residents during personal care interactions with nursing assistants.	England	Behavioural management and communication-skills training program was provided to the nursing staff.	The duration of the baseline and post-training care observed by Computer-assisted behavioral observation for 10 mins.	66 residents (52 women and 14 men) with mean age 80.8	Mini-Mental State Exam	The quality of the interactions between certified nursing assistant and residents was modified. Significant effects were seen as positive statements of nursing staff were increased and the percentage of agitation during personal care interactions were reduced ($p < 0.01$).	9

Sherratt et al., 2004	NHS dementia service	To examine the impact of social interaction in music on challenging behaviour of people with dementia.	England	Taped commercial music, taped music and live music played by a musician.	Three months	24 participants. Male= 14 Female= 10 Age from 53–89 year	Psion and Mini<br><br>Mental State Examination (MMSE)	Live music was effective in increasing levels of well-being significantly ($p<0.01$). However, No significant differences found for challenging behaviours.	07
Berg et al., 1998	Ward of dementia care.	To reveal nurses understanding about patients in dementia care	Sweden	Open-ended unstructured interview was conducted in intervention time.	Nurses were interviewed entire year (total 24 interviews conducted altogether).	13 nurses and a ward housed 11 patients were included in the study.	Phenomenological-hermeneutic analysing method.	Strategies revealed to manage patients with verbal agitative behaviour. No scores were provided for the verbal disruptive behaviour.	7
